# Biochemical Studies on Human Ornithine Aminotransferase Support a Cell-Based Enzyme Replacement Therapy in the Gyrate Atrophy of the Choroid and Retina

**DOI:** 10.3390/ijms25147931

**Published:** 2024-07-19

**Authors:** Gioena Pampalone, Davide Chiasserini, Francesca Pierigè, Emidio Camaioni, Pier Luigi Orvietani, Alessandro Bregalda, Michele Menotta, Ilaria Bellezza, Luigia Rossi, Barbara Cellini, Mauro Magnani

**Affiliations:** 1 Department of Medicine and Surgery, University of Perugia, P.le L. Severi 1, 06132 Perugia, Italy; gioena.pampalone@unipg.it (G.P.); davide.chiasserini@unipg.it (D.C.); pierluigi.orvietani@unipg.it (P.L.O.); ilaria.bellezza@unipg.it (I.B.); 2 Department of Biomolecular Sciences, University of Urbino Carlo Bo, 61029 Urbino, Italy; francesca.pierige@uniurb.it (F.P.); alessandro.bregalda@uniurb.it (A.B.); michele.menotta@uniurb.it (M.M.); mauro.magnani@uniurb.it (M.M.); 3 Department of Pharmaceutical Sciences, University of Perugia, Via del Liceo 1, 06122 Perugia, Italy; emidio.camaioni@unipg.it

**Keywords:** ornithine aminotransferase, gyrate atrophy, pyridoxal phosphate, enzyme administration, drug delivery, erythrocytes

## Abstract

The gyrate atrophy of the choroid and retina (GACR) is a rare genetic disease for which no definitive cure is available. GACR is due to the deficit of ornithine aminotransferase (hOAT), a pyridoxal 5′-phosphate-dependent enzyme responsible for ornithine catabolism. The hallmark of the disease is plasmatic ornithine accumulation, which damages retinal epithelium leading to progressive vision loss and blindness within the fifth decade. Here, we characterized the biochemical properties of tetrameric and dimeric hOAT and evaluated hOAT loaded in red blood cells (RBCs) as a possible enzyme replacement therapy (ERT) for GACR. Our results show that (i) hOAT has a relatively wide specificity for amino acceptors, with pyruvate being the most suitable candidate for ornithine catabolism within RBCs; (ii) both the tetrameric and dimeric enzyme can be loaded in RBC retaining their activity; and (iii) hOAT displays reduced stability in plasma, but is partly protected from inactivation upon incubation in a mixture mimicking the intracellular erythrocyte environment. Preliminary ex vivo experiments indicate that hOAT-loaded RBCs are able to metabolize extracellular ornithine at a concentration mimicking that found in patients, both in buffer and, although with lower efficiency, in plasma. Overall, our data provide a proof of concept that an RBC-mediated ERT is feasible and can be exploited as a new therapeutic approach in GACR.

## 1. Introduction

**Figure 1:**
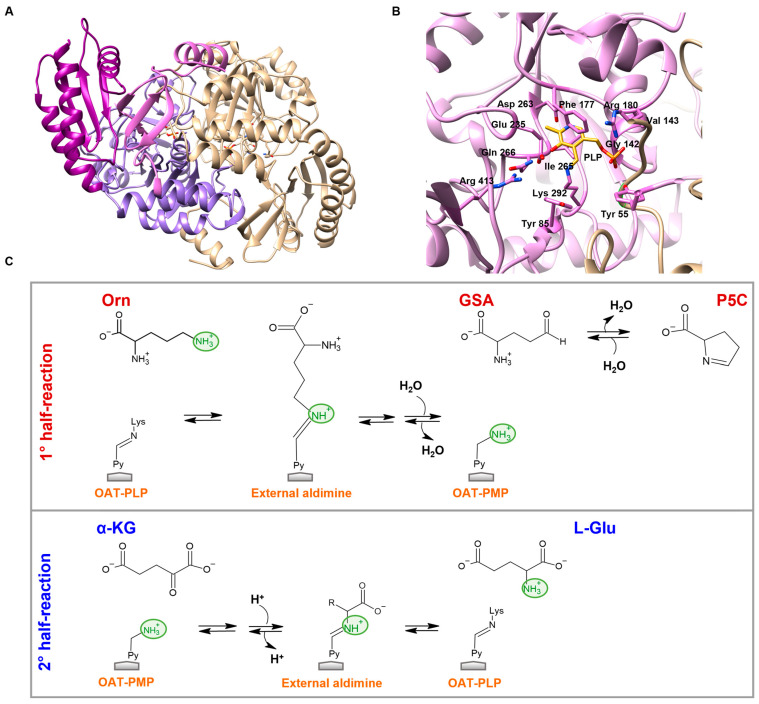
Structural features and catalytic pathway of hOAT. (**A**) The three-dimensional structure of the hOAT dimeric unit (pdb:1OAT) with one monomer colored light yellow and the neighboring monomer colored as follows: N-terminal domain, pink; large domain, violet; C-terminal domain, magenta. N and O atoms are colored blue and red, respectively. (**B**) Details of the active site of hOAT. PLP anchored through the Schiff base linkage with Lys292 is shown as yellow sticks. The residues located in the active site at 5 angstroms from PLP are shown as pink sticks and labeled. (**C**) The hOAT transaminase reaction mechanism.

Human ornithine aminotransferase (hOAT) is a mitochondrial pyridoxal 5′-phosphate (PLP)-dependent enzyme mainly expressed in the liver, kidney, intestine, and retina, and involved in the arginine/polyamine pathway and the glutamate/proline pathway [1]. The structural features of hOAT are those typical of the Fold Type I family of PLP-enzymes, with each subunit comprising an N-terminal domain, a large domain, and a small C-terminal domain (Figure 1A). In solution, the enzyme shows a tetrameric assembly [2], although site-directed mutagenesis studies demonstrate that the functional unit of the enzyme is the dimer. Indeed, the mutation of the interfacial residue Arg217 to alanine generates a variant (OAT-R217A) that is stable in solution as a dimer and shows biochemical features identical to those of the wild-type [3]. In each monomer, PLP is bound to Lys292 through a Schiff base linkage and stabilized by a number of non-covalent interactions with active site residues (Figure 1B).

hOAT catalyzes the conversion of L-ornithine (Orn) and α-ketoglutarate to glutamate semialdehyde (GSA), which spontaneously cyclizes to the proline precursor pyrroline-5-carboxylate, and glutamate [3]. As shown in Figure 1C, the first half-reaction is the δ-transamination of L-ornithine, which converts hOAT from the PLP-form to the pyridoxamine 5′-phosphate (PMP)-form, while the second half-reaction is the α-transamination of α-ketoglutarate, which regenerates the PLP-form and produces glutamate. Crystallographic studies have indicated that Arg180, Tyr55, Glu235, and Arg413 at the active site play a pivotal role in the differential orientation of the two substrates [4,5]. Moreover, recent kinetic analyses using different amino donors have shown that the δ-amino group of Orn is important in promoting catalysis [6]. However, the amino acceptor specificity of hOAT has never been investigated.

The functional deficit of hOAT leads to an increase in Orn plasmatic levels, the main hallmark of the gyrate atrophy of the choroid and retina (GACR). GACR is a rare autosomal recessive disease, whose clinical symptomatology consists of progressive vision loss as a consequence of the toxic effect of hyperornithinemia on retinal cells, and, in particular, on retinal pigmented epithelium cells [7,8]. Several mutations have been identified and characterized, and they cause the hOAT deficit by interfering with catalysis and/or folding [9,10,11]. The treatment options available for patients with GACR are poor and mainly consist of an arginine-restricted diet, which aims at reducing the burden of Orn to delay the onset of the symptoms but is associated with poor patient compliance. Moreover, a beneficial effect of Vitamin B6 administration has been reported in a few patients [12,13,14].

Based on the evidence that a reduction in the amount of plasmatic Orn is associated with better preservation of the visual function in GACR [15,16], we reasoned that the intravenous administration of functional hOAT could be exploited as enzyme replacement therapy (ERT) to counteract hyperornithinemia. ERT is based on the administration of a functional enzyme to correct the underlying defect that causes a monogenic rare disease [17,18,19]. It is worth noting that the main problem of ERT is the short in vivo benefit due to the fast removal of the administered enzyme from circulation and/or the inactivation of the same by elicited anti-enzyme antibodies, even upon surface protection through PEGylation [20,21]. To overcome these limits, red blood cells (RBCs) represent an ideal carrier to be exploited for the administration of recombinant hOAT as a therapeutic enzyme. The main factors that make RBCs an ideal carrier for a therapeutic enzyme are (i) their biocompatibility and non-immunogenicity, (ii) their long in vivo life span, and (iii) their ability to protect the entrapped enzyme from rapid clearance and from inactivating antibodies [22]. Furthermore, a number of safe methods to produce erythrocyte-based drug-delivery systems are actually available and exploited in clinical investigations [23,24].

Here, we performed an in-depth analysis of the biochemical properties of tetrameric and dimeric hOAT aiming at identifying possible alternatives and more suitable acceptors of the Orn amino group, as well as at investigating the general thermodynamic stability of the two enzymes under conditions mimicking blood circulation. We then set up the hOAT loading in human RBCs and tested their ability to behave as bioreactors and metabolize extracellular Orn in ex vivo experiments. The data obtained provide the proof of concept that an ERT based on the use of RBCs as carriers for hOAT is feasible, and can counteract blood hyperornithinemia in patients with GACR.

## 2. Results and Discussion

### 2.1. OAT-wt and OAT-R217A Can Use Different Amino Acceptors for Orn Transamination

**Scheme 1:**
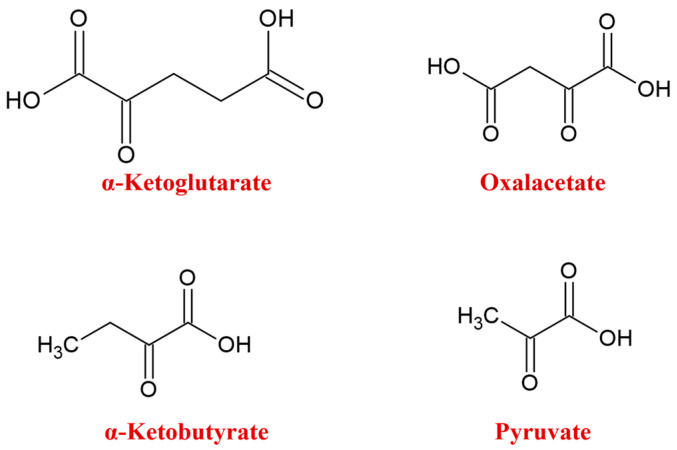
Chemical structure of hOAT amino acceptors.

Focusing on the possibility to implement RBCs loaded with hOAT as bioreactors to metabolize plasmatic Orn, we obtained recombinant purified OAT-wt and OAT-R217A enzymes with a high yield and suitable purity using an available expression and purification protocol [3]. Indeed, as the amino acceptor is a co-substrate necessary for hOAT-mediated Orn metabolism, we reasoned that to maintain activity within RBCs upon the consumption of the physiological substrate α-KG, the enzyme should use alternative compounds. Since hOAT displays a certain level of promiscuity for amino donor substrates [6], we investigated if the enzyme might be able to catalyze Orn transamination using alternative α-ketoacids. We evaluated three potential substrates sharing structural and/or chemical similarities with α-KG: oxalacetate, whose structure is analogous to that of α-KG but with a 4-carbon chain; α-ketobutyrate, which shares with α-KG the α-keto group but lack the distal carboxylic group; and its lower homolog pyruvate, a key metabolic ketoacid (Scheme 1).

**Figure 2:**
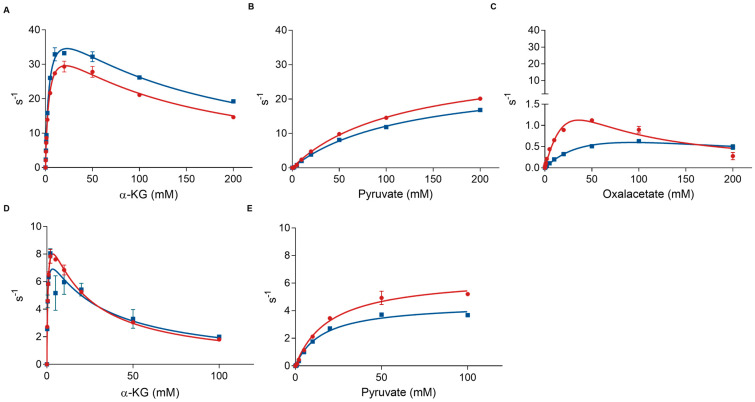
Kinetic parameters of OAT-wt and OAT-R217A for Orn transamination in the presence of different amino acceptors. The kinetic parameters for OAT-wt (red dots) and OAT-R217A (blue dots) in the presence of 100 mM Orn and increasing concentrations of (**A**) α-KG, (**B**) pyruvate, and (**C**) oxalacetate in 50 mM Hepes buffer, pH 8.0, 150 mM NaCl at 25 °C. (**D**,**E**) show the kinetic parameters of OAT-wt (red dots) and OAT-R217A (blue dots) in the presence of 1 mM L-Orn and increasing concentrations of (**D**) α-KG and (**E**) pyruvate in 50 mM Hepes buffer, pH 8.0, 150 mM NaCl at 25 °C. The enzyme activity is expressed as nmol product/s/nmol E (s^−1^). The blue and red lines represent the fitting of the data to the Michaelis/Menten equation or a modified equation taking into account substrate inhibition (see Section 3).

We determined the kinetic parameters for each amino acceptor at both saturating Orn concentration and 1 mM Orn, the latter condition mimicking the plasmatic concentration found in patients with GACR (Figure 2 and Table 1). In addition to the physiological substrate α-KG, both OAT-wt and OAT-R217A are able to catalyze Orn transamination using pyruvate and oxaloacetate as amino acceptors, although the kinetic behavior in the presence of the various co-substrates shows some differences. Indeed, both α-KG and oxalacetate give rise to substrate inhibition (Figure 2A,C,D), a behavior not observed in the presence of pyruvate (Figure 2B,E). Notably, neither OAT-wt nor OAT-R217A showed any transaminase activity in the presence of α-ketobutyrate in a classical assay mixture. Nevertheless, upon extensive incubation at high protein concentration in the presence of saturating Orn and 40 mM α-ketobutyrate, a time-dependent production of P5C could be measured, from which we estimated a rate of 0.045 ± 0.001 s^−1^ and 0.032 ± 0.003 s^−1^ for OAT-wt and OAT-R217A, respectively, values approximately 1000-fold lower as compared with those of α-KG.

To confirm this hypothesis with a more experimental basis, we compared the interaction of the PMP-forms of OAT-wt and OAT-R217A with α-KG and α-ketobutyrate through spectroscopic analyses. As expected, the PMP-form of both enzymes shows an absorbance band at 333 nm due to the binding of the aminic form of the coenzyme. Upon the addition of α-KG or α-ketobutyrate, the conversion of the 333 nm band to the PLP-form absorbing at 420 nm is observed. However, while in the presence of the physiological substrate α-KG, the process is completed within the mixing time (Figure S1A,B); in the presence of α-ketobutyrate, time-dependent changes are observed and the process occurs with an apparent rate constant of 0.061 ± 0.007 min^−1^ and 0.082 ± 0.006 min^−1^ for OATwt and OAT-R217A, respectively (Figure S1C,D). It must be underlined that these values are lower than the estimated rate of the overall transamination of the Orn/α-ketobutyrate pair. This could be due to either PMP dissociation from the apoenzyme, or the concomitant occurrence of the reverse transamination reaction on the product α-aminoketobutyrate, as previously reported for other PLP-dependent aminotransferases [25].

The kinetic parameters of OAT-wt and OAT-R217A for Orn transamination in the presence of each amino acceptor allow us to observe that (i) both enzymatic forms show the highest *k*_cat_ and the lowest apparent K_m_ for the physiological substrate α-KG, which, however, gives rise to inhibition at high millimolar concentrations; (ii) a one-carbon reduction in the chain length in oxaloacetate causes a more than 90% reduction in catalytic efficiency, driven both by a reduced *k*_cat_ and by an increased apparent K_m_, but it does not significantly affect the K_i_ value; (iii) the three-carbon ketoacid pyruvate shows the lowest affinity for the enzyme (as indicated by the highest K_m_), but displays a *k*_cat_ value of the same order of magnitude with respect to that of α-KG, with no evident inhibition effects. A qualitatively similar behavior is observed at sub-saturating Orn concentrations, although, as expected for enzymes using a ping-pong catalytic mechanism, the apparent K_m_ and K_i_ values for α-ketoacids are significantly lower. From these results, it can be confirmed that both OAT-wt and OAT-R217A are able to use alternative amino acceptors such as pyruvate or oxalacetate to perform Orn transamination. Notably, the intracellular concentrations of α-KG reported in the literature is 5 µM against a plasma level of 40 µM [26,27]. Moreover, the dialysis step performed during RBC loading causes a partial depletion of small molecules, thus probably implying a further decrease in the intracellular α-KG concentration (see Section 2.3). On the other hand, the expected intracellular concentrations of pyruvate in RBCs is 70–200 µM [28], and the RBC loading procedure involves a last step of resealing performed in the presence of 100 mM pyruvate [29]. Therefore, we can hypothesize that pyruvate could play a major role in Orn transamination by OAT-loaded RBCs. To obtain insights into the role of pyruvate as an amino acceptor under conditions that better mimic those of patients with GACR, we determined the kinetic parameters at 37 °C using 1 mM Orn as an amino donor (Table S2). We found the expected increase in *k*_cat_, but also an increase in K_m_. The resulting catalytic efficiency at 37 °C is of the same order of magnitude as that measured at 25 °C (0.3 and 0.28 s^−1^mM^−1^ for OAT-wt and OAT-R217A, respectively), thus further supporting pyruvate as a good candidate involved in Orn transamination in OAT-loaded RBCs.

The obtained data also allow us to draw conclusions about the mechanistic properties of hOAT. Indeed, it is known that the enzyme must recognize and differently bind the two substrates Orn and α-KG so that a δ-transamination occurs on the first, while an α-transamination occurs on the second [4,5,6,30,31]. The structure of the enzyme in a complex with Orn analogs shows four residues that play a key role in this process: (i) Arg180 and Tyr55 anchor the α-COOH and the α-NH_2_ groups of L-Orn, respectively, thus promoting the correct binding of the substrate, whose δ-NH_2_ group must face the Schiff base in a proper position for proton abstraction by Lys292 [4]; (ii) Glu235 interacts through an ion pair with Arg413, thus preventing its possible interaction with the α-COOH of L-Orn that would generate a non-productive complex. Indeed, the substitution of Arg180 with a Thr residue prevents the proper Orn collocation at the active site, allowing an alternative binding mode prone to undergo α-transamination [5]. Unfortunately, no structural data on the α-KG binding mode are available due to unsuccessful crystallization attempts attributed to the reversible nature of the α-KG/Glu transamination reaction [4]. Nevertheless, it has been hypothesized that Arg180 interacts with the γ-COOH group, while a conformational switch of Glu235 upon α-KG binding allows Arg413 binding to the α-COOH group so that the keto acid results in a suitable position for α-transamination [5].

**Figure 3:**
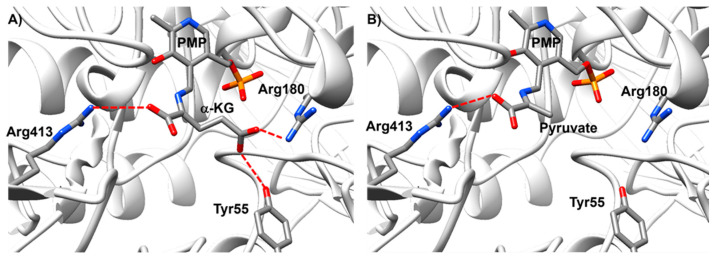
Predicted binding poses of the ketimine PMP-α-KG (**A**) and PMP-pyruvate (**B**). All the structures are shown in sticks (colored by element type); the salt bridge interactions and selected hydrogen bonds with Tyr55 are drawn as dashed red lines.

A molecular docking study was then performed aiming at providing structural insights and possibly explaining the different kinetic parameters for the α-transamination of the four ketoacids under study. To this aim, we manually constructed the four different ketimine intermediates and docked them into the hOAT active site. Indeed, hOAT co-crystallized with the inhibitor (3S,4R)-3-amino-4-(difluoromethyl)cyclopent-1-ene-1-carboxylate [32] was instrumental for this aim. In this structure, the inhibitor is covalently linked to the PMP cofactor and Arg413 is in a more proper extended conformation. As a result, we obtained the predicted binding poses employing Autodock-GPU version 1.5 [33] as an accurate molecular docking software (see Section 3.2.4) of all the ketimines within the binding site A, as depicted in Figure 3. The predicted poses of the most populated clusters of the four ketimines (Figure S2) are placed with the α-carboxyl moiety towards Arg413, thus mimicking the correct positioning for the α-transamination reaction (Figure 1, second half-reaction). Furthermore, the PMP-bound substrates are walled by the two aromatic amino acids Tyr85 and Phe177. In the case of oxalacetate and α-KG, the distal COOH goes towards Arg180 and Tyr55. However, only α-KG is able, in the predicted pose, to form a further salt bridge interaction with Arg180 (distance about 3 Å, Figure 3A). On the other hand, the shorter chain and the absence of the distal COOH in pyruvate does not allow a similar stabilization (Figure 3B). These results agree with the lower K_m_ of oxalacetate and α-KG as compared with pyruvate, and with the fact that α-KG probably binds in a position more suitable for C4′-proton abstraction. Furthermore, although the estimated binding energies are quite similar among the four ligands, the rank order of these energies for α-KG, oxalacetate, and pyruvate ketimines correlates with the experimental affinities, expressed as K_m_, of the substrates (Table 1 and Table S3). As for the α-ketobutyrate ketimine intermediate, the putative pose docking energy places the chain in a position analogous to that of the other substrates, with no apparent steric clashes (Figure S2). Therefore, the structural reasons underlying the low enzymatic activity of hOAT toward the latter substrate remain unclear. It can be only speculated that the longer side chain as compared with pyruvate gives rise to a conformation in solution that does not allow the perfect positioning of either the proton bound to the C4′ of the coenzyme, which must be oriented perpendicularly to the PLP ring as predicted by the Dunathan hypothesis [34], or Lys292, which must be in a position suitable to deprotonate C4′ and reprotonate the Cα of the substrate [2,4].

The finding that some amino acceptors give rise to substrate inhibition merits some considerations. Structural and kinetic data [35,36] and more recent results on the interaction of hOAT with alternative amino donors [6] have highlighted the possibility of a double binding mode of amino acidic substrates based on the presence/absence of the Glu235-Arg413 salt bridge. Indeed, an analysis of the kinetic data we obtained in the presence of the different amino donors allows us to observe that the ketoacids endowed with proximal and distal COOH groups (i.e., α-KG and oxaloacetate) display substrate inhibition effects. Taking into account the predicted best pose of α-KG, it can be hypothesized that the presence of the two COOH groups allows two alternative binding modes at the active site: one “productive” with the α-COOH in a proper position for α-proton abstraction and C4′ reprotonation; one “non-productive”, with the α-COOH bound in a distal position (e.g., toward Arg180) and characterized by a lower affinity. This view would explain why pyruvate shows a low affinity for the enzyme (because just one COOH group stabilizes the ligand at the active site), but it does not give rise to inhibition effects. Finally, it should be mentioned that substrate inhibition is only evident at non-physiological concentrations of α-KG and oxaloacetate. Nonetheless, this behavior provides interesting insights into the molecular mechanisms that govern hOAT’s ability to “recognize” the substrate and perform a δ-transamination or an α-transamination. The importance of this aspect for the ability to detoxify Orn is demonstrated by the finding that the pathogenicity of GACR-associated mutations that alter the active site conformation can compromise the productive binding of the substrate, thus preventing proper catalysis [5].

### 2.2. General Stability Studies of hOAT

**Figure 4:**
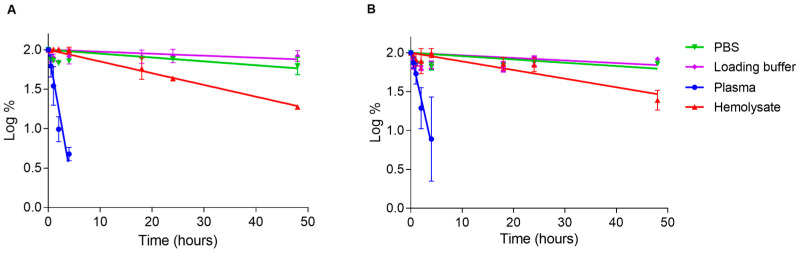
General stability of OAT-wt and OAT-R217A. The time-dependent changes in enzymatic activity of OAT-wt (**A**) and OAT-R217A (**B**) in loading buffer (purple), PBS (green), hemolysate (red), and plasma (blue) upon incubation at 37 °C in the presence of 100 µM exogenous PLP. The data represent the relative activity of the enzyme, expressed as the logarithm of nmoles of product/s/ nmole enzyme percentage vs. incubation time, expressed as hours. The data represent the mean ± SEM (*n* = 3).

Since loaded RBCs are expected to work for a long period of time at 37 °C, we carried out experiments aiming at evaluating the general stability of OAT-wt and OAT-R217A in these experimental conditions. We monitored the time-dependent changes in Orn transaminase activity up to 48 h incubation under four different conditions: PBS (as a negative control), 10 mM Hepes pH 7.4 plus 154 mM NaCl plus 5 mM glucose (the loading buffer), hemolysate (to mimic the intracellular microenvironment of the loaded enzyme), and plasma (as a positive control to mimic the condition of an unloaded enzyme). The data reported in Figure 4 reveal that OAT-wt and OAT-R217A, respectively, (i) display an estimated half-life of 67 ± 13 h and 78 ± 18 h upon incubation in PBS or 140 ± 41 h and 100 ± 31 h upon incubation in loading buffer; (ii) show a half-life reduced to 0.89 ± 0.05 h and 1.0 ± 0.1 h in the plasma, which increases to 23 ± 1 h and 30 ± 2 h in hemolyzed RBCs. In Western blot analyses, we did not detect any loss of protein under any of the incubation media (Figure S3), thus indicating that the activity reduction is not due to proteolytic events. Moreover, since the enzyme undergoes a 20-fold dilution in the assay mixture, the possibility that the observed loss of activity is due to the presence of molecules behaving as OAT inhibitors in the plasma and in the RBC hemolysate seems unlikely. Therefore, the most likely hypothesis is the occurrence of aspecific modification events and/or the aggregation of the protein. Indeed, the high stability of OAT-wt and OAT-R217A in buffered solutions implies that events affecting solubility such as denaturation or aspecific protein/protein interactions involving surface-charged groups are limited under these conditions [37]. The very low stability of intracellular proteins when incubated in plasma is not uncommon, as already observed for other enzymes considered candidates for enzyme administration therapies, such as α-galactosidases for Fabry disease [38] or phenylalanine ammonia lyase for phenylketonuria [39], thus suggesting that the intracellular environment could keep the two proteins protected from time-dependent inactivation. Therefore, we can conclude that although tetrameric and dimeric hOAT show limited stability in plasma, the finding that stability is improved upon incubation in a lysate of RBCs prompts for the encapsulation inside erythrocytes as a suitable strategy to preserve functionality in circulation.

### 2.3. RBC Loading with Recombinant OAT-wt and OAT-R217A

**Figure 5:**
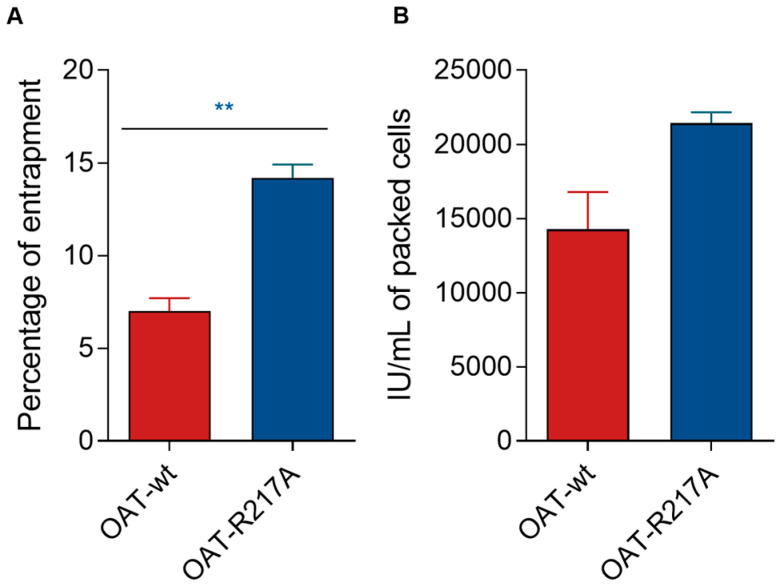
Loading efficiency of OAT-wt and OAT-R217A. (**A**) Percentage of entrapment in RBCs for OAT-wt and OAT-R217A. (**B**) Enzyme activity of OAT-wt and OAT-R217A upon RBC loading. ** *p* < 0.01 vs. OAT-wt as control.

We evaluated the feasibility of a cell-mediated therapy for GACR based on RBCs used as bioreactors through ex vivo experiments aiming at evaluating the ability of RBCs loaded with tetrameric or dimeric hOAT to consume extracellular Orn. Briefly, the loading procedure consists of a hypotonic dialysis followed by isotonic resealing and reannealing. In the first step, isolated human RBCs at a hematocrit of 70% were placed in a dialysis tube in the presence of 3.90 ± 0.17 mg of tetrameric hOAT (specific activity 16.9 U/mg) or 3.20 ± 0.01 mg dimeric hOAT (specific activity 20.9 U/mg) and dialyzed against a hypotonic solution to allow the opening of the membrane pores that favors the encapsulation of hOAT by a passive mechanism. In the second step, an isotonic resealing was performed to enable the closure of the pores and, consequently, the entrapment of the enzyme within the RBCs. The enzymatic activity was measured on the hOAT-loaded RBCs and the percentage of entrapment was evaluated. The data reported in Figure 5 show that OAT-R217A displays an entrapment two-fold higher as compared to the wild-type protein (14.2% OAT-R217A vs. 7.0% OAT-wt). Taking into account Native PAGE experiments indicating that the oligomeric state of the two proteins does not change upon incubation in the hypotonic or isotonic resealing solutions (Figure S4), it can be concluded that the higher entrapment fold of OAT-R217A is probably due to the smaller size of the dimer that favors internalization. Unexpectedly, the activity of dimeric hOAT expressed as IU/mL packed RBCs measured upon encapsulation in RBCs was not significantly different with respect to that of the tetrameric counterpart. Considering that the overall stability of OAT-R217A is comparable to that of OAT-wt, this behavior cannot be attributed to protein degradation. Rather, it can be speculated that it depends on the presence of presently unknown components that possibly partly reduce the activity of the loaded protein.

RBCs loaded with either OAT-wt or OAT-R217A were then incubated for 48 h in a Hepes-buffered solution or in plasma in the presence of 1 mM Orn to mimic in vitro the hyperornithinaemia of patients with GACR. The loaded RBCs kept at 37 °C for 48 h mostly maintained their integrity, showing only 10 ± 2% hemolysis at the last incubation time compared to time 0 in both Hepes solution and plasma.

**Figure 6:**
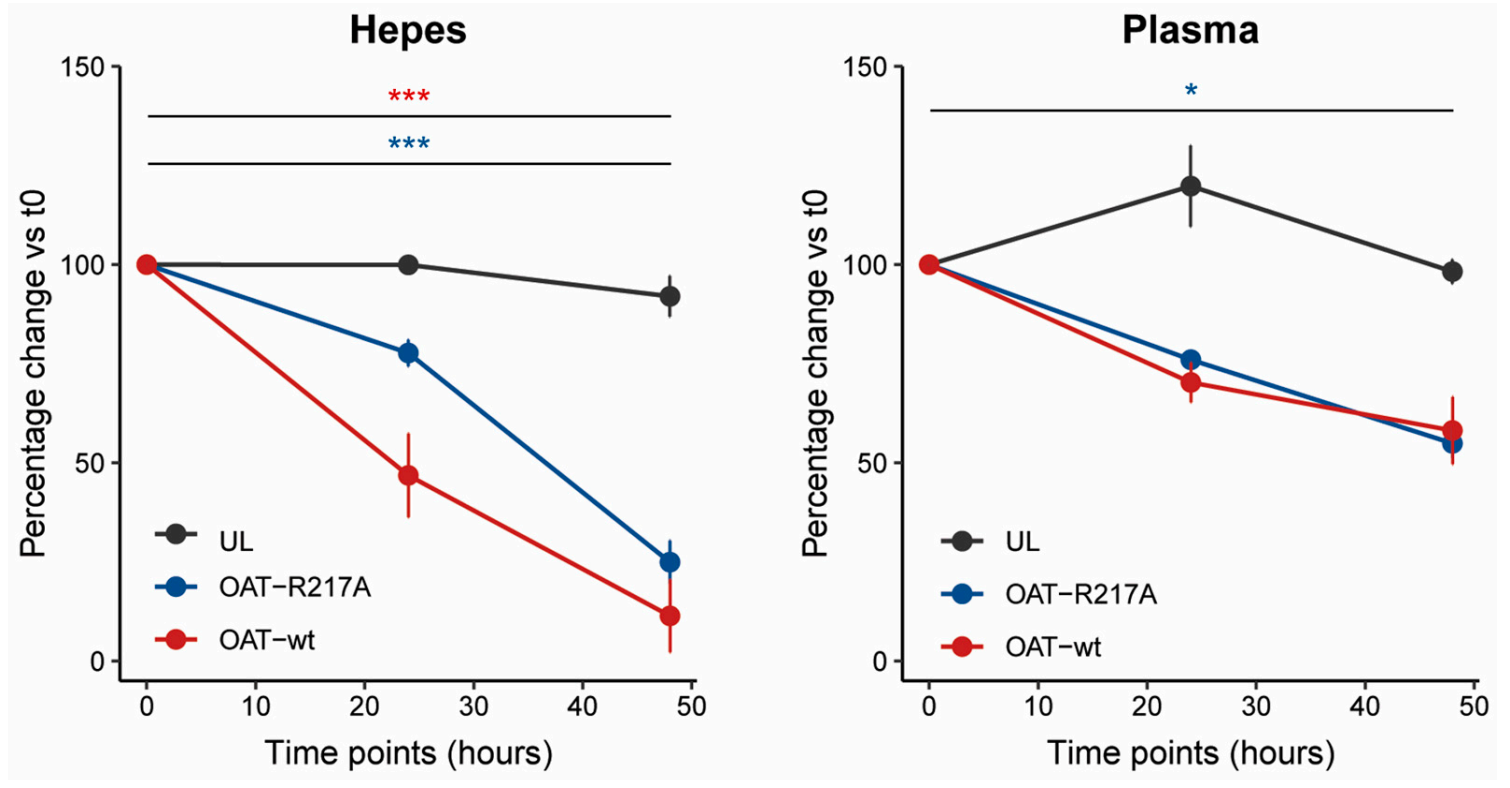
Residual Orn concentration as measured by HPLC in Hepes and plasma after incubation with loaded RBCs. The RBCs loaded with OAT-wt and OAT-R217A as well as the unloaded RBCs (UL) as control were incubated with 1 mM Orn in Hepes buffer or in plasma as indicated. The residual Orn in the supernatant was measured by the HPLC analysis and expressed as % change vs. t0. The data represent the mean ± SEM (*n* = 3). * *p* < 0.05 vs. t0; *** *p* < 0.001 vs. t0.

Aliquots of each mixture were withdrawn at 24 h and 48 h, the cells were pelleted, and the residual Orn present in the supernatant was evaluated through the HPLC analysis upon deproteinization and derivatization. As reported in Figure 6, no changes in the extracellular Orn levels were observed with the unloaded RBCs both in Hepes buffer and in plasma. On the other hand, the incubation of the loaded RBCs in Hepes buffer led to a time-dependent consumption of Orn, which reached 90% after 48 h incubation. A qualitatively similar behavior was observed upon incubation in human plasma, although the efficiency of the consumption was lower, reaching approximately 50% after 48 h. A comparison of the data obtained with the RBCs loaded with OAT-wt and those loaded with OAT-R217A did not reveal significant differences, in agreement with the similar specific activity of the RBCs loaded with the two enzymatic forms.

**Figure 7:**
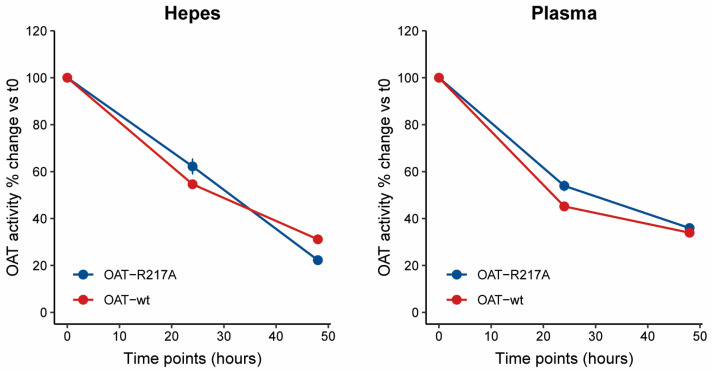
Residual activity measured on loaded RBCs during incubation in Hepes buffer or plasma. Enzyme transaminase activity under each condition is expressed as % change vs. t0. Data represent mean ± SEM (*n* = 2).

To investigate the occurrence of any change in the enzymatic activity of the encapsulated proteins during RBC incubation, we measured the residual hOAT activity in the RBC pellet at each time point. The data reported in Figure 7 show that a remarkable time-dependent reduction in enzyme activity occurs both in the Hepes buffer and in plasma, in agreement with the measured half-life of the two proteins upon incubation in an RBC lysate (Figure 4). Since the loss of activity shows a similar trend in the two conditions, it cannot explain the non-complete consumption of the extracellular Orn in plasma (Figure 6). Based on the published data [40] one possibility that can be advanced is that a factor in plasma, perhaps a specific protein, controls Vitamin B6 distribution between plasma and RBCs, thus preventing the intracellular transport of PLP. This would progressively impair Orn catabolism, thus possibly justifying the lower activity of enzyme-loaded RBCs in metabolizing Orn when incubated in plasma supplemented with 100 µM PLP. Another hypothesis is that the lower efficiency in plasma is due to competition for the CAT-1 transporter, which displays a K_m_ of 0.1–0.16 mM [41] and could be saturated with competing cationic amino acids when Orn concentration drops below a certain level. Finally, the possible binding of Orn to plasmatic proteins should be taken into consideration, because it would reduce the levels of unbound Orn free to be metabolized by loaded RBCs in plasma. In this regard, the ability of serum albumin to bind cationic compounds has been widely demonstrated [42]. The future characterization of the products of Orn metabolism by hOAT-loaded RBCs will possibly help in better understanding the process in plasma and how to shift the equilibrium toward a complete Orn consumption.

## 3. Materials and Methods

### 3.1. Materials

PLP, L-Orn, α-KG, α-ketobutyrate, oxaloacetate, pyruvate, 2-aminobenzaldehyde, dimethyl sulphoxide (DMSO), isopropyl-β-d-thiogalactopyranoside (IPTG), phenylmethylsulfonyl fluoride (PMSF), leupeptin, and pepstatin were purchased from Sigma (St. Louis, MO, USA). All the other chemicals were of the highest purity available.

### 3.2. Methods

#### 3.2.1. Expression and Purification of OAT-wt and OAT-R217A

*E. coli* BL21 cells were transformed with the constructs pET43a Δ26-439 OAT-wt and pET43a Δ26-439 OAT-R217A encoding the mature form of human OAT-wt and the OAT-R217A strikethrough variant, respectively. The cells were grown in 2 L of Luria broth with 50 mg/L ampicillin at 37 °C to an absorbance of 0.4–0.6 at 600 nm. Protein expression was induced by the addition of 0.5 mM IPTG and by decreasing the temperature to 30 °C for 15 h. The cells were harvested by centrifugation at 4300× *g* for 10 min at 4 °C and resuspended in 20 mM sodium phosphate (NaP) buffer pH 7.6 (buffer A), 50 μM PLP, protease inhibitor cocktail (Roche, Basel, Switzerland), 500 μM PMSF, and 0.1 mM EDTA. Lysozyme was then added to a concentration of 0.2 mg/mL and the culture was incubated for 20 min at room temperature. After freeze/thaw, leupeptin (0.5 μg/mL) and pepstatin (0.7 μg/mL) were added and the suspension was centrifuged at 18,000× *g* for 30 min at 4 °C. The supernatant was recovered, adjusted to pH 7.6, and loaded on a DEAE HiPrep FF 16/10 column equilibrated with buffer A at 2 mL/min. A linear gradient of 0–100% of 150 mM NaP pH 7.6 in 240 mL elution volume was then applied. Under these conditions, OAT elutes from the column between 115 and 150 mM NaP. The fractions showing the presence of catalytically active OAT were pooled and reconstituted with 100 μM PLP. The solution was then concentrated using Amicon Ultra 15 units (Merck Millipore, Tullagreen, Carrigtwohill, Co Cork, Ireland) and applied to a HiPrep 16/60 Sephacryl S-100 High-Resolution column equilibrated in 50 mM Hepes, pH 7.4, 500 mM NaCl. The eluted protein was concentrated using Amicon Ultra 15 devices (Merck Millipore, Tullagreen, Carrigtwohill, Co Cork, Ireland) to a concentration lower than 200 μM and stored at −20 °C. This method yields about 40 mg and 30 mg of purified OAT-wt and OAT-R217A, respectively, per liter of bacterial culture.

#### 3.2.2. Activity Assay

The OAT activity assay is based on the formation of glutamate semialdehyde, which spontaneously generates pyrroline-5-carboxylate (P5C). The reaction between P5C and 2-aminobenzaldehyde (2-AB) produces a derivate complex absorbing at 440 nm. 50 nM purified proteins (or 10, 25, and 50 µL of cellular lysate at 5% Hct) were incubated in a reaction mixture containing 100 μM PLP, 50 mM α-KG, and 100 mM L-Orn in 50 mM Hepes buffer pH 8.0, 150 mM NaCl in a final volume of 200 μL. The mixture was then incubated at 25 °C for 10 min and the enzymatic reaction was stopped by the addition of trichloroacetic acid (TCA) 10% *v*/*v*. To detect the formation of P5C, 100 µL of 2-aminobenzaldehyde 3.6 mg/mL (previously resuspended into 70% HCl 1 M and 30% DMSO) were added to the mixture followed by incubation at 25 °C for 40 min in the dark. At the end of incubation, the assay mixture was centrifuged at 13,000× *g* for 2 min and the absorbance at 440 nm of the supernatant containing the complex 2AB-P5C was measured. To calculate the amount of P5C formed during the reaction, a molar extinction coefficient of 2710 M^−1^cm^−1^ was used.

To determine the kinetic parameters for different amino acceptors (α-KG, α-ketobutyrate, oxaloacetate, and pyruvate), purified OAT-wt or OAT-R217A (50 nM–500 nM) were incubated in the presence of 100 μM PLP, L-Orn 100 mM in 50 mM Hepes buffer, pH 8.0, NaCl 150 mM at 25 °C or 37 °C at various substrate concentration (from 0.25 to 200 mM) as reported above. The data of nmol of product/s/nmol enzyme as a function of substrate concentration were fitted using the Michaelis/Menten equation (Equation (1)) or a modified equation that takes into account substrate inhibition (Equation (2)) [43]

(1) $$Y=V_{max}*X/(K_{m}+X)$$

(2) $$Y=V_{max}*X/(K_{m}+X*\left(\frac{1+X}{K_{i}}\right))$$

where *V_max_*, *K_m_*_,_ and *K_I_* represent the maximum enzyme velocity, the substrate concentration that yields a half-maximal velocity, and the dissociation constant for substrate binding in such a way that two substrates can bind to an enzyme, respectively.

#### 3.2.3. Stability Assays

The general stability of OAT-wt and OAT-R217A was monitored upon the incubation of each enzyme (1 µM) at 37 °C in the presence of 100 µM PLP under four different conditions: 10 mM Hepes pH 7.4 plus 154 mM NaCl plus glucose 5 mM (loading buffer), PBS, hemolysate erythrocytes, and plasma. Hemolysate erythrocyte is prepared through a 1:1 dilution of the RBC pellet in water [44]. At various time points (up to 48 h), 10 μL of the incubation mixture was used to perform an activity assay. The assay mixtures containing 10 µL of each incubation mixture were used for a standard activity assay. The data were plotted as logarithms of the percentage residual activity as a function of time and were fitted to a linear regression curve and the half-life values were calculated using the equation

(3) T12=log⁡50−2m

where *T_1_*_/*2*_ and *m* represent the half-life and the slope value obtained from linear regression curve fit, respectively.

In addition, to monitor any time-dependent loss of protein, 2.5 μg of OAT-wt and OAT-R217A were loaded on 10% SDS–PAGE and transferred on a nitrocellulose membrane. To rule out any gross difference in gel loading, the membrane was visually inspected upon Ponceau staining. The membrane was immunoblotted with anti-OAT antibody (OriGene, Rockville, MD, USA) (1:1000) in 5% (*w*/*v*) milk in TTBS (50 mM Tris–HCl, pH 7.5, 150 mM NaCl, 0.1% Tween 20) overnight at 4 °C. After three washes in TTBS, the membrane was incubated with HRP-conjugated anti-mouse IgG (1:5000) in 5% milk in TTBS for 1 h at room temperature. Immunocomplexes were visualized by an enhanced chemiluminescence kit (ECL, Pierce Biotechnology, Rockford, IL, USA).

#### 3.2.4. Molecular Docking Studies

Molecular modeling calculations were performed on an Intel-based workstation (i9, 20CPUs) with a NVIDIA GeForce RTX 4060 Ti graphic card.

The dimeric assembly of hOAT with 2 symmetrical binding sites was analyzed: The 3D coordinates were retrieved from the Protein Data Bank (PDB ID: 7TEV [32]) resolution of 1.91 Å, biological assembly-1. AutoDockTools (ADT, ver. 1.5.7), a chemistry file translation program [45], was then used to convert the pdb into the corresponding pdbqt-type file by removing water molecules and other ligands, and, finally, by adding polar hydrogens and Gasteiger partial charges. The three-dimensional structure of the four studied substrates bound to PMP (ketimines intermediates with ionized carboxylates) were built and optimized through Avogadro (ver. 1.2) [46] and saved as a mol2 file, and imported into the ADT where partial charges were added, non-polar hydrogens merged, and routable bonds defined, affording a new pdbqt-type file. All the docking simulations were then performed with AutoDock-GPU (version 1.5), an OpenCL-accelerated version of the widely used AutoDock 4.2.6 that is also CUDA compatible. The search algorithm in AutoDock-GPU is a Lamarckian Genetic Algorithm (LGA) hybridized with the gradient-based local search ADADELTA [33]. A total of 100 runs for each docking simulation were employed. In each run, a population of 150 individuals with 42,000 generations and 2,500,000 energy max evaluations were employed. Operator weights for crossover and mutation were set to 80% and 2%, respectively. The binding site A, used by autogrid4 for the calculations of maps (parameter file: ADA.1 bound.dat), was defined as a cubic box using a grid of 40 × 40 × 40 points each with a grid space of 0.375 Å centered at coordinates x = −17.3y = −7.9z = 0.6. All the other parameters were set as default values. Cluster analyses were performed on the docking results using an RMS tolerance of 2.0 Å. Finally, the most populated with the lowest binding energy clusters were considering for analysis by ADT and, finally, the resulting docking complexes were evaluated and rendered with UCSF Chimera (ver. 1.17) [47]. All the molecular modeling files are available from the authors upon request.

#### 3.2.5. Spectroscopic Measurements

The PMP-form of OAT-wt and OAT-R217A was produced upon the incubation of the enzyme with 100 mM Orn in Hepes 50 mM, pH 7.4, NaCl 500 mM followed by forced dialysis with the same buffer without Orn. Absorbance spectra were registered using a Jasco V-750 spectrophotometer (JASCO Corporation 2967-5 Ishikawamachi Hachioji-shi Tokyo Japan) using 1 cm path length quartz cuvettes at a protein concentration of 5–10 μM in 50 mM Hepes, pH 7.4, 500 mM NaCl. OAT-wt and OAT-R217A concentrations were determined from the absorbance at 280 nm using the molar absorption coefficient of 124,767 M^−1^cm^−1^. PLP content was measured after the treatment of the holoenzyme with 0.1 M NaOH using the molar absorption coefficient of 6600 M^−1^cm^−1^ of free PLP at 388 nm.

#### 3.2.6. Loading Procedure

We performed the RBC loading procedure with both recombinant OAT-wt and OAT-R217A according to Magnani et al. [29], with some modifications. Human blood was collected from healthy donors using heparinized tubes by the Transfusion Centre of the “S. Maria della Misericordia” Hospital in Urbino (PU). Briefly, to develop hOAT-loaded erythrocytes for in vitro studies, recombinant purified OAT was encapsulated into human RBCs by means of a procedure including (i) hypotonic dialysis (hypotonic buffer: 10 mM NaH_2_PO_4_ pH 7.4, 10 mM NaHCO_3_, 20 mM glucose, 2 mM ATP, 3 mM GSH, and 100 μM PLP) in the presence of the protein to be encapsulated resulting in the homogeneous distribution of the enzyme within and outside the cell (at +4 °C), (ii) “isotonic resealing and reannealing” using a hyperosmotic solution (10% *v*/*v* Pigpa: 100 mM inosine, 20 mM ATP, 10 mM glucose anhydrous, 100 mM sodium pyruvate, 4 mM MgCl_2_, 190 mM NaCl, 1.6 M KCl, and 33 mM NaH_2_PO_4_) to restore isotonicity (at +37 °C) with the closure of the pores and the consequent encapsulation of the target protein. After the resealing and reannealing steps, the loaded erythrocytes were washed twice in the loading buffer and added with 100 µM PLP to remove the residues of the hypertonic solution, the lysate RBCs, and the unentrapped enzyme. Finally, the packed, loaded RBCs were resuspended in the loading buffer (10 mM Hepes pH 7.4 plus 154 mM NaCl plus 5 mM glucose) containing 100 µM PLP at about 40% Ht, and the amount of the entrapped enzyme was evaluated on the RBC lysates, as detailed above.

#### 3.2.7. Native PAGE Gel Electrophoresis

Native PAGE electrophoresis was performed by loading 10 µg of the purified protein mixed with 2× dye (25% glycerol, 62.5 mM Tris-HCl pH 6.8, and 1% Bromophenol blue) and (i) hypotonic buffer (10 mM NaH_2_PO_4_ pH 7.4, 10 mM NaHCO_3_, 20 mM glucose, 2 mM ATP, 3 mM GSH, and 100 μM PLP); (ii) hyperosmotic solution (10% *v*/*v* Pigpa: 100 mM inosine, 20 mM ATP, 10 mM glucose anhydrous, 100 mM sodium pyruvate, 4 mM MgCl_2_, 190 mM NaCl, 1.6 M KCl, and 33 mM NaH_2_PO_4_); (iii) loading buffer (10 mM Hepes pH 7.4 plus 154 mM NaCl plus 5 mM glucose plus 100 µM PLP). The gel was run in Tris/glycine buffer (25 mM Tris/HCl, 192 mM glycine, pH 8.3) at a constant voltage of 100 V until the dye front was released. The gel was then stained with Coomassie Brilliant Blue R-250 (PanReac AppliChem, Darmstadt, Germany) and visualized using an iBright imager (Thermo Fisher, Waltham, MA, USA).

#### 3.2.8. Orn Metabolism by Loaded RBCs

The evaluation of Orn consumption by the loaded RBCs in the ex vivo study was performed by incubating the erythrocytes at +37 °C for up to 48 h in the loading buffer or plasma, both containing 1 mM Orn, 100 µM α-KG, and 100 µM PLP to a final 10% Ht. At planned time points (0, 24, and 48 h), 400 μL RBC suspension was centrifuged, and the supernatants were immediately frozen at −80 °C for the Orn evaluation by the HPLC analysis while the RBC-packed cells were resuspended in the loading buffer added with 100 µM PLP and immediately frozen at −80 °C for the evaluation of the hOAT enzymatic stability. The unloaded RBCs (UL, i.e., the cells subjected to the encapsulation procedure without enzyme) were used as a negative control.

#### 3.2.9. HPLC Analysis

Orn was measured by HPLC upon derivatization with 1-Fluoro-2,4-dinitrobenzene (DNFB) according to [48,49]. A total of 90 µL of supernatants derived from RBC loaded with OAT-wt or OAT-R217A was deproteinized by using 30 µL of TCA 10% and frozen for 20 min at −20 °C. The samples were then centrifuged at 13,000× *g* at 4 °C for 10 min. The supernatant was recovered and treated with NaOH 2N to adjust the pH to 7.0. The samples were freeze-dried and the residual dry pellets were resuspended in borate buffer 0.2 M pH 9.0. As an internal control, 100 µM Norvaline was added to each sample in a final volume of 50 μL. After the addition of 25 μL of DNFB 72 mM to each mixture, the samples were incubated at 60 °C for 1 h in the dark. The reaction was then stopped on ice by adding 175 μL of cold PBS pH 7.0 (final volume 250 µL). Twenty microliter of deproteinized and derivatized samples were then loaded on an EC NUCLEODUR 100-5 C18 (250 × 4.6 mm) Machery-Nagel column at 45 °C connected to a JASCO LC-4000 HPLC control system. The mobile phases were as follows: A, 40 mM NaH_2_PO_4_ buffer pH 7.8; B, 45/45/10% ACN/MetOH/water. The conditions used for gradient elution are reported in Table S1, and the run was performed at a flow rate of 1 mL/min. A Jasco UV-4070 detector set at 360 nm was employed. The peak corresponding to Orn was integrated using the ChromNAV software (version 2.4.0.5). A standard curve of peak area was prepared using commercially available Orn.

#### 3.2.10. Statistical Analysis

The experiments were performed at least in triplicate and in any case, the standard error mean (SEM) was less than 10%. The statistical analysis and plotting were performed using GraphPad Prism Version 6.0 (GraphPad software, San Diego, CA, USA) and R-software version 2.7.0 [50]. The experiments were performed at least in triplicate. The data are reported as the mean ± standard error of the mean (SEM). Two group comparisons were performed with a T-test, while the evaluation of ornithine consumption was carried out using two-way ANOVA. A *p*-value < 0.05 was considered significant for all the analyses.

## 4. Conclusions

An efficient and specific therapy for GACR is not currently available [14]. The main hallmark of the disease is hyperornithinemia consequent to the hOAT deficit, a condition that damages retinal cells causing progressive vision loss. In this work, we have expanded the knowledge on the kinetic properties of hOAT, and we have explored the possibility of developing an ERT using RBCs as bioreactors to degrade plasmatic Orn through the encapsulation of functional hOAT, an already validated therapeutic strategy for a number of rare diseases [51].

The main findings that support the feasibility of an RBC-mediated ERT in GACR are the following: (1) The kinetic properties of OAT-wt and of a dimeric variant endowed with similar functional properties (OAT-R217A) reveal that both forms can utilize alternative amino acceptors such as pyruvate and oxalacetate. This aspect is very important since Orn catabolism by hOAT requires the presence of sufficient amounts of α-ketoacid acceptor. Among those tested, pyruvate is the most promising for the cell-mediated ERT, because it does not give rise to inhibition effects, and allows it to reach high catalytic efficiency at the RBC intracellular concentration. (2) Tetrameric and dimeric hOAT show very low stability in plasma but are protected from inactivation under conditions that could mimic encapsulation in RBCs. This implies that loading is a good procedure to improve the in vivo half-life of the enzyme. (3) In ex vivo experiments, human RBCs encapsulated with OAT-wt or OAT-R217A metabolize extracellular Orn at concentrations similar to those found in patients with GACR. Although the process is less efficient in plasma where a complete Orn consumption cannot be reached, the results obtained provide a strong proof of principle that RBCs loaded with hOAT can metabolize extracellular Orn. In this regard, it must also be considered that an approximately 50% reduction in plasmatic Orn levels is sufficient to protect retinal tissues from damage in mice models of GACR [15]. Moreover, a non-complete Orn consumption will maintain the physiological metabolism of this amino acid. Nonetheless, possible limitations must be taken into account. First, the full activity of loaded hOAT can be achieved only if sufficient amounts of the coenzyme PLP and of α-ketoacid co-substrate are present. As for the coenzyme, the treatment of patients with GACR with Vitamin B6 can increase the intracellular concentration of PLP high enough to keep the enzyme in the holo form functional for transamination [11,52,53]. Moreover, the last step of the loading procedure involves a resealing performed in the presence of 100 mM pyruvate [23], which can behave as a co-substrate for Orn catabolism, as demonstrated by our results. Second, P5C produced in OAT-loaded RBC is a reactive metabolite, previously shown to be linked to oxidative stress and aging, as well as to behave as a Vitamin B6 inactivator [54,55,56]. However, one of the enzymes able to metabolize P5C is P5C reductase that produces proline. Three different genes encoding P5C reductase are present in the human genome, one of which (codified by the gene PYCR3) produces a cytoplasmic enzyme [57]. This enzyme is probably responsible for the activity measured in erythrocytes already several years ago [58] and more recently, was identified as part of the RBC proteome [59]. Indeed, P5C can be used by RBCs to boost the pentose phosphate pathway and produce intermediates that promote purine recycling [60]. Therefore, it is possible to hypothesize that excess P5C is processed by this enzyme, although further experiments will be necessary to fully understand the metabolism of ornithine-derived P5C in OAT-loaded RBCs. Finally, in ex vivo experiments, loaded hOAT displays very low stability and undergoes a significant loss of activity. Thus, the possibility to engineer the protein to increase its thermodynamic stability once loaded in RBCs must be considered.

Nevertheless, although in vivo experiments will be necessary to validate this strategy, our data represent an important premise for the future development of a new therapeutic strategy for GACR.

## Figures and Tables

**Table 1:** Kinetic parameters for OAT-wt and OAT-R217A in the presence of Orn and different amino acceptors.

		Orn 100 mM			Orn 1 mM	
		α-KG	Pyruvate	Oxalacetate	α-KG	Pyruvate
**OAT-wt**	***k*_cat_ (s^−1^)**	40 ± 1	32 ± 1	2.0 ± 0.2	11.0 ± 0.3	6.5 ± 0.2
	**K_m_ (mM)**	3.8 ± 0.2	116 ± 6	19 ± 3	0.67 ± 0.05	20 ± 2
	**K_I_ (mM)**	122 ± 9	/	105 ± 22	18 ± 1	/
**OAT-R217A**	***k*_cat_ (s^−1^)**	46 ± 1	27 ± 1	1.5 ± 0.3	8.5 ± 0.8	4.5 ± 0.2
	**K_m_ (mM)**	3.7 ± 0.2	121 ± 7	67 ± 17	0.4 ± 0.1	16 ± 2
	**K_I_ (mM)**	141 ± 11	/	128 ± 40	30 ± 9	/

## Data Availability

The original contributions presented in the study are included in the article/supplementary material, further inquiries can be directed to the corresponding author.

## Supplementary Materials

The following supporting information can be downloaded at: https://www.mdpi.com/article/10.3390/ijms25147931/s1.
