# Supplementary material for: Biochemical Studies on Human Ornithine Aminotransferase Support a Cell-Based Enzyme Replacement Therapy in the Gyrate Atrophy of the Choroid and Retina
Source: Int J Mol Sci. 2024 Jul 19;25(14):7931. doi: 10.3390/ijms25147931 (PMC11277095; doi:10.3390/ijms25147931)
Supplement: Supplementary file 1 [file ijms-25-07931-s001.zip › ijms-3050154-supplementary.pdf]

Biochemical Studies on Human Ornithine Aminotransferase Support a Cell-based Enzyme Replacement Therapy in the Gyrate Atrophy of the Choroid and Retina

Gioena Pampalone [1a], Davide Chiasserini [1a], Francesca Pierigè [2], Emidio Camaioni [3], Pier Luigi Orvietani [1], Alessandro Bregalda [2], Michele Menotta [2], Ilaria Bellezza [1], Luigia Rossi [2*], Barbara Cellini [1*], and Mauro Magnani [2]

[1] Department of Medicine and Surgery, University of Perugia, P.le L. Severi 1, 06132 Perugia, Italy
[2] Department of Biomolecular Sciences, University of Urbino Carlo Bo, 61029 Urbino, Italy
[3] Department of Pharmaceutical Sciences, University of Perugia, Via del Liceo 1, 06122 Perugia, Italy
* Correspondence: B.C., barbara.cellini@unipg.it; L.R., luigia.rossi@uniurb.it

[a]These authors contributed equally

Supplementary Information

Table S1: Composition of the gradient elution used for the HPLC analyses on the derivatized samples. A: mobile phase A; B: mobile phase B.

Time (min)	A %	B %
0.5	90	10
3	60	40
5	60	40
10	55	45
15	50	50
20	45	55
25	40	60
27	0	100
31	0	100
32	90	10
39	90	10

Table S2: Kinetic parameters for OAT-wt and OAT-R217A for pyruvate at 37°C in the presence of 1 mM Orn.

	OAT-wt	OAT-R217A
k_{cat} (s^{-1})	9.7 ± 0.4	10.7 ± 0.3
K_m (mM)	80 ± 8	51 ± 3
k_{cat}/K_m ($mM^{-1}s^{-1}$)	0.12 ± 0.01	0.21 ± 0.04

Table S3: Autodock-GPU docking results of the ketimine intermediates in the binding site of OAT.

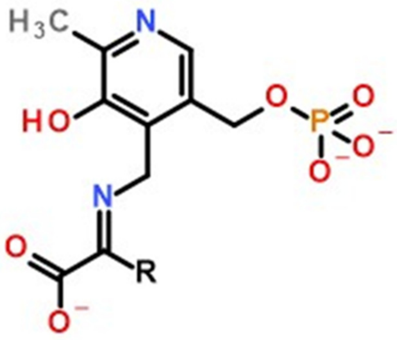

#	Ketimines R =	Lowest-ranked docking energies of the more populated clusters (cluster population)
PMP-αKG	$CH_2CH_2COO^-$	-9.1 Kcal/mol (26 out of 100 runs)
PMP-oxalacetate	CH_2COO^-	-8.7 Kcal/mol (24 out of 100 runs)
PMP-αketobutyrate	CH_2CH_3	-8.6 Kcal/mol (36 out of 100 runs)
PMP-pyruvate	CH_3	-8.5 Kcal/mol (44 out of 100 runs)

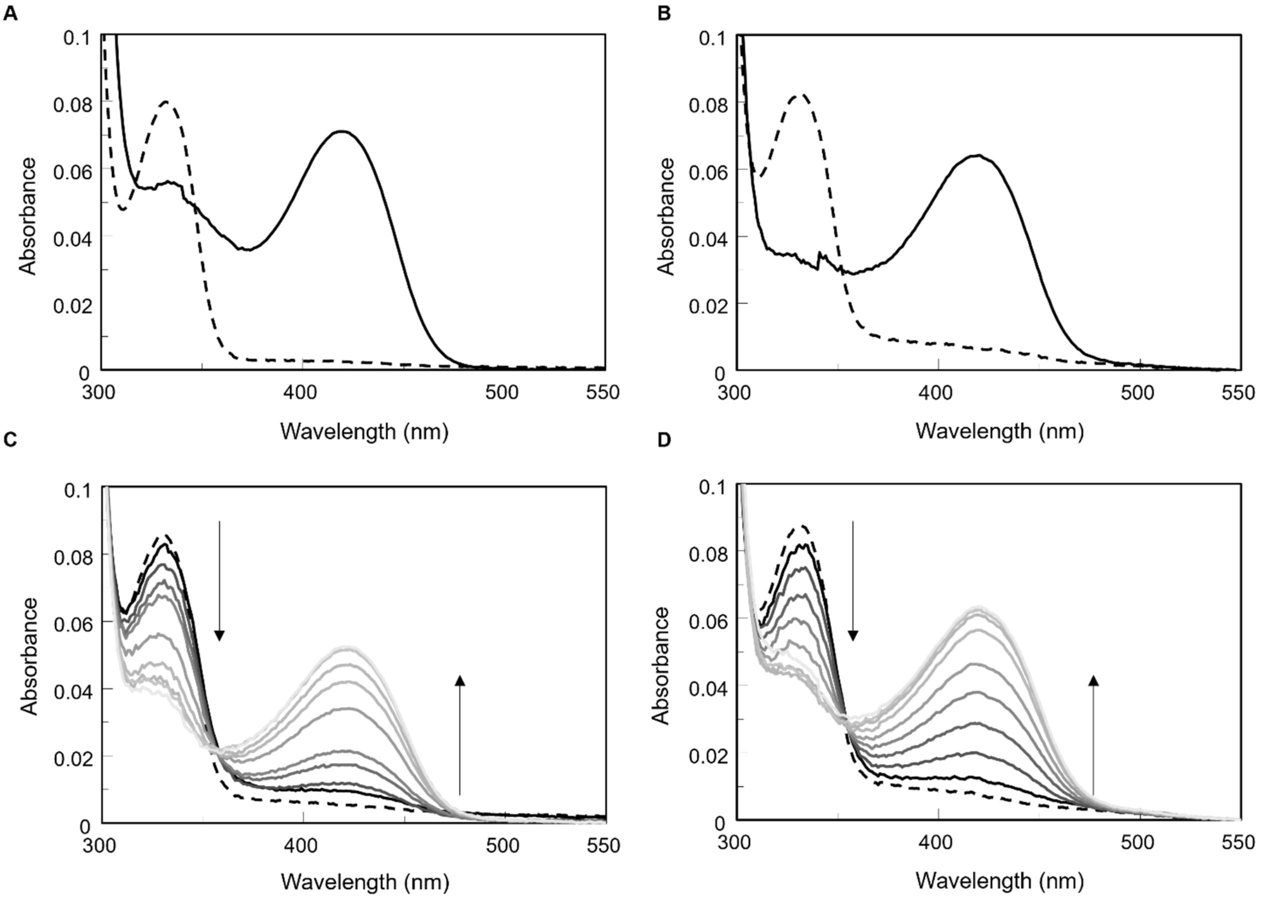

Figure S1: Spectral changes in OAT-PMP upon the addition of α-KG or α-Ketobutyrate. The time-dependent spectral changes in 7 μM OAT-wt (**A**) and OAT-R217A (**B**) in the absence (dotted lines) or in the presence (black lines) of 40 mM α-KG. The time-dependent spectral changes in 7 μM OAT-wt (**C**) and OAT-R217A (**D**) in the absence (dotted lines) or in the presence (grey lines) of 40 mM α-Ketobutyrate. The arrows indicate the absorbance changes. The spectra have been registered at 2, 4, 7, 9, 16, 22, 28, 37, and 45 minutes for OAT-wt and at 2, 4, 8, 11, 14, 21, 29, 36, and 45 min for OAT-R217A. The experiments have been performed in buffer 50 mM Hepes, pH 8.0, 150 mM NaCl at 25°C.

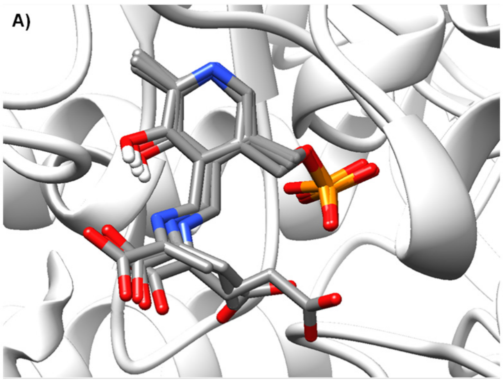

Figure S2. Predicted lowest energy binding poses of the more populated cluster (see Table S3) ketimines of hOAT with ketoacids under study into the catalytic site A of OAT. The structures are shown in sticks (colored by element type).

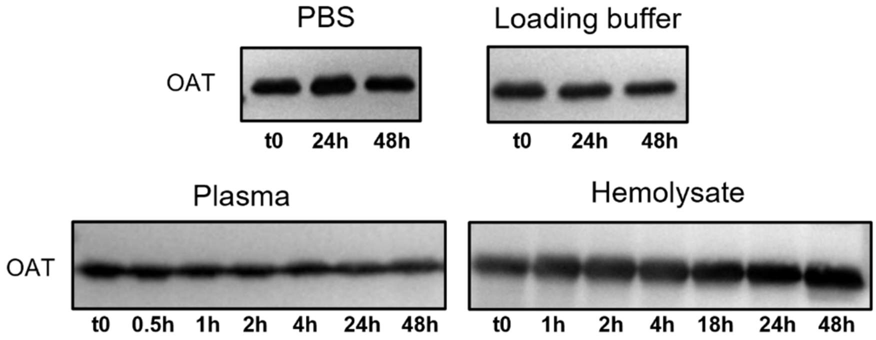

Figure S3: SDS-PAGE (PBS and loading buffer) and Western blot (plasma and hemolysate) analyses performed on 0 hOAT upon incubation under the indicated conditions at 37°C in the presence of 100 μM exogenous PLP. The amount of protein in each lane was 5 μg in SDS-PAGE and 0.2 μg in Western blot analyses.

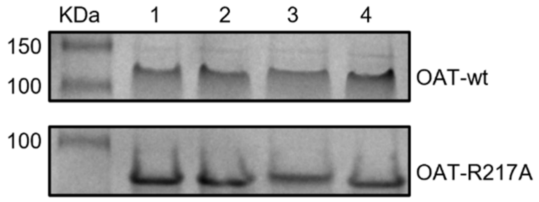

Figure S4: Native PAGE analysis of OAT-wt and OAT-R217A in buffer conditions used during RBC loading. Lanes are coded as follows: KDa, molecular weight markers; 1, control in storage buffer; 2, hypotonic buffer (10 mM NaH_2PO_4 pH 7.4, 10 mM $NaHCO_3$, 20 mM glucose, 2 mM ATP, 3 mM GSH, and 100 μM PLP); 3, hyperosmotic solution (10% v/v Pigpa: 100 mM inosine, 20 mM ATP, 10 mM glucose anhydrous, 100 mM sodium pyruvate, 4 mM $MgCl_2$, 190 mM NaCl, 1.6 M KCl, and 33 mM NaH_2PO_4); 4, loading buffer (10 mM Hepes pH 7.4 plus 154 mM NaCl plus 5 mM glucose plus 100 μM PLP).
